# Mental distress among young people in inner cities: the Resilience, Ethnicity and AdolesCent Mental Health (REACH) study

**DOI:** 10.1136/jech-2020-214315

**Published:** 2021-02-08

**Authors:** Gemma Knowles, Charlotte Gayer-Anderson, Stephanie Beards, Rachel Blakey, Samantha Davis, Katie Lowis, Daniel Stanyon, Aisha Ofori, Alice Turner, Schools Working Group, Vanessa Pinfold, Ioannis Bakolis, Ulrich Reininghaus, Seeromanie Harding, Craig Morgan

**Affiliations:** 1 Health Service and Population Research, King's College London, London, UK; 2 ESRC Centre for Society and Mental Health, King's College London, London, UK; 3 National Childrens Bureau, London, UK; 4 Centre for Society and Mental Health, King's College London, London, UK; 5 The McPin Foundation, London, UK; 6 Centre for Implementation Science, Health Services and Population Research Department, King's College London, London, UK; 7 Department of Biostatistics and Health Informatics, King's College London, London, UK; 8 Department of Public Mental Health, Central Institute of Mental Health, Medical Faculty Mannheim, University of Heidelberg, Mannheim, Germany; 9 Department of Nutritional Sciences, King's College London, London, UK

**Keywords:** mental health, social epidemiology, social inequalities, public health, child health

## Abstract

**Background:**

Recent estimates suggest around 14% of 11–16 years in England have a mental health problem. However, we know very little about the extent and nature of mental health problems among diverse groups in densely populated inner cities, where contexts and experiences may differ from the national average.

**Aims:**

To estimate the extent and nature of mental health problems in inner city London, overall and by social group, using data from our school-based accelerated cohort study of adolescent mental health, Resilience, Ethnicity and AdolesCent Mental Health.

**Methods:**

Self-report data on mental health (general mental health, depression, anxiety, self-harm) were analysed (n, 4353; 11–14 years, 85% minority ethnic groups). Mixed models were used to estimate weighted prevalences and adjusted risks of each type of problem, overall and by gender, cohort, ethnic group and free school meals (FSM) status.

**Results:**

The weighted prevalence of mental health problems was 18.6% (95% CI 16.4% to 20.8%). Each type of mental health problem was more common among girls compared with boys (adjusted risk ratios: mental health problems, 1.33, 95% CI 1.18 to 1.48; depression, 1.52, 1.30 to 1.73; anxiety, 2.09, 1.58 to 2.59, self-harm, 1.40, 1.06 to 1.75). Gender differences were more pronounced in older cohorts compared with the youngest. Mental health problems (1.28, 1.05 to 1.51) and self-harm (1.29, 1.02 to 1.56)—but not depression or anxiety—were more common among those receiving (vs not receiving) FSM. There were many similarities, with some variations, by ethnic group.

**Conclusions:**

Adolescent mental health problems and self-harm are common in inner city London. Gender differences in mental health problems may emerge during early adolescence.

## Introduction

Mental ill health is a major public health concern.^1^ Adolescence is a critical period of emotional and behavioural development and the period during which most adult mental health problems first emerge.^2^ Adolescents who develop mental health problems, particularly persistent or recurring problems, are at increased risk of wide-ranging adverse outcomes in adulthood.^3–5^ The costs are vast. In 2009/2010, the estimated economic and social costs of mental ill health in England was £105.2 billion.^6^

The most recent national survey of the Mental Health of Children and Young People in England (MHCYP) suggests around 14% of 11–16 years have a mental health problem.^7^ This is broadly in line with other national surveys using self-report measures, for example, 12% in Understanding Society, 2011/2012.^8^ Despite widespread perceptions that mental health problems have risen among young people in recent years, the MHCYP surveys suggest little change in the overall prevalence of mental health problems among 5–15 years over the past 15–20 years. Notable increases in emotional problems were evident among older (16–19 years) females, but among younger adolescents (11–16 years) there was at most a small increase in emotional problems between 1999 and 2017 and little change for other types of mental health problems.^7^ This is somewhat surprising in the context of profound social change in the UK during this period (eg, recession, austerity, changes in migration). However, the effects of such changes are unlikely to be uniform across social groups and geographical areas. While national surveys provide useful information on the prevalence of mental health problems nationally, they do not provide detailed information on specific groups in which prevalence may differ from the national average, for example, minority ethnic and socially disadvantaged groups in densely populated inner cities. Moreover, national surveys may obscure changes over time in some groups. At present, there is very little up-to-date information on the extent and nature of problems among young people from diverse groups and inner city areas in the UK. Limited evidence suggests young people from UK minority ethnic groups generally have similar or better mental health than their white British peers, but findings are inconsistent, the most robust data are now almost two decades old,^9 10^ and much of the recent evidence is based on small, unrepresentative samples and data aggregated over broad, heterogeneous groups (eg, ‘Black’, ‘Asian’).^7 11^ One of the more consistent findings is that young people from Indian or, in some studies, ‘Asian’ backgrounds tend to have better mental health than their white British peers.^11 12^

In this paper, we present baseline data from a prospective study of adolescent mental health in inner city London—Resilience, Ethnicity and AdolesCent Mental Health (REACH)—on the extent and nature of mental health problems among adolescents from diverse backgrounds. REACH is the largest localised contemporary study of its kind in the UK. We hypothesised that the prevalence of mental health problems would be higher in inner city London compared with nationally; higher among girls compared with boys, particularly depression; higher among those from economically disadvantaged compared with more affluent backgrounds; and lower among those from Indian/Pakistani/Bangladeshi backgrounds but similar across other ethnic groups.

## Methods

### Study design

REACH is an accelerated cohort study of adolescent mental health in two inner city London boroughs, Lambeth and Southwark. Three cohorts—age 11–12 (cohort 1; school year 7), 12–13 (cohort 2; year 8) and 13–14 (cohort 3; year 9) at baseline—are completing annual questionnaires. A subset (around 20%) are also completing face-to-face interviews and cognitive assessments.

### Setting, participants

Participants were recruited from 12 mainstream secondary schools in the two boroughs. These boroughs are among the most densely-populated, socioeconomically and ethnically diverse areas in England,^13–15^ and have high rates of adult mental health problems.^16^ Schools were selected using a quota sampling approach. All eligible schools were stratified based on the proportion of pupils receiving free school meals (FSM) and the proportion from minority ethnic groups, and we used existing contacts to fill the quotas. Schools were approached until quotas were filled. This approach generated a sample that was highly representative of the target population based on key demographic characteristics. Of the schools approached, one declined to take part due to time constraints. At the 12 participating schools, all students in years 7–9 were invited to participate (n, 4945).

### Procedures

Informed consent was obtained for all participants. Eligible young people attended an in-school talk about REACH, delivered by a researcher, and received written information for themselves and their parents/carers. Parents were asked to return a form, or contact the school or research team, if they did not want their child to take part. On the day of assessment, students received further information from researchers and provided written assent before completing a battery of validated questionnaires, in-class, on study tablet computers. The baseline assessment took around 1 hour to complete and collected detailed information on mental health and putative risk and protective factors. Trained researchers were present throughout all sessions to explain procedures and answer questions. All baseline questionnaires were administered between February 2016 and January 2018.

### Mental health

General mental health was assessed using the 25-item self-report Strength and Difficulties Questionnaire (SDQ) for 11–17 years,^17^ a widely used and validated^17^ measure of emotional and behavioural problems during the previous 6 months. Scores ≥18 indicate high-to-very high risk of mental health problems (hereafter, ‘mental health problem’). Internalising and externalising scores were calculated using established procedures.^18^ Higher scores indicate more difficulties.

### Depression

Depression was assessed using the 13-item Short Mood and Feelings Questionnaire (SMFQ),^19^ a self-report measure of depressive symptoms over the previous 2 weeks. Scores ≥12 indicate high risk of depression (hereafter, ‘depression’). The SMFQ has high internal consistency and convergent validity and moderate diagnostic accuracy among adolescents.^19 20^

### Anxiety

Generalised anxiety was assessed using the 7-item self-report Generalised Anxiety Disorder Scale (GAD-7), which measures experiences of generalised anxiety over the preceding 2 weeks.^21^ Scores ≥10 indicate moderate-to-severe anxiety (hereafter, ‘anxiety’).^21^ Recent community-based research supports the validity and reliability of the GAD-7 among adolescents.^22^

### Self-harm

Lifetime self-harm (yes/no) was self-reported using an item from the Development and Adolescent Well-being Assessment: ‘Over the whole of your lifetime, have you ever tried to harm or hurt yourself?’^23^

### Demographic information

Participants were asked to describe their ethnic group based on eighteen categories used in the 2011 ONS census.^24^ We combined some smaller groups (eg, Arab, Chinese) and used ten ethnic groups in this analysis (table 1). FSM status, a marker of household income, was self-reported.

**Table 1:** Participant characteristics

	All (n, 4353)		Boys (n, 2138)		Girls (n, 2215)		Cohort 1 (n, 1593)		Cohort 2 (n, 1421)		Cohort 3 (n, 1339)	
	N	%	N	%	N	%	N	%	N	%	N	%
Gender (n, 4353)												
Boys	2138	49.1	··	··	··	··	778	48.8	701	49.3	659	49.2
Girls	2215	50.9	··	··	··	··	815	51.2	720	50.7	680	50.8
Cohort (school year group) (n, 4353)												
1 (Y7)	1593	36.6	778	36.4	815	36.8	··	··	··	··	··	··
2 (Y8)	1421	32.6	701	32.8	720	32.5	··	··	··	··	··	··
3 (Y9)	1339	30.8	659	30.8	680	30.7	··	··	··	··	··	··
Eligible for free school meals (n, 4113)												
No	3137	76.3	1563	77.2	1574	75.4	1142	76.5	1015	75.4	980	76.9
Yes	976	23.7	461	22.8	515	24.7	351	23.5	331	24.6	294	23.1
Ethnic group (n, 4353)												
Black African	1113	25.6	545	25.5	568	25.6	383	24.0	374	26.3	356	26.6
Black Caribbean	719	16.5	350	16.4	369	16.7	234	14.7	257	18.1	228	17.0
Other black	127	2.9	51	2.4	76	3.4	49	3.1	46	3.2	32	2.4
Mixed white and black	380	8.7	182	8.5	198	8.9	143	9.0	117	8.2	120	9.0
Other mixed ethnic groups	237	5.4	105	4.9	132	6.0	87	5.5	82	5.8	68	5.1
Indian, Pakistani, Bangladeshi	181	4.2	83	3.9	98	4.4	79	5.0	57	4.0	45	3.4
Latin American	217	5.0	110	5.1	107	4.8	58	3.6	79	5.6	80	6.0
White British	667	15.3	337	15.8	330	14.9	285	17.9	185	13.0	197	14.7
Non-British white	409	9.4	213	10.0	196	8.9	166	10.4	121	8.5	122	9.1
Any other/unknown	303	7.0	162	7.6	141	6.4	109	6.8	103	7.3	91	6.8

The proportion of white British students was slightly higher in Cohort one compared with cohorts 2 and 3 (χ^2^=40.3223, df=18, p=0.002).

### Statistical analysis

Multilevel linear and logistic regression models (melogit and mixed commands) were used to estimate weighted prevalence (margins command) of each type of mental health problem, overall and by group (cohort, gender, ethnic group, FSM), accounting for clustering within schools. The marginalised delta method was used to calculate risk ratios (RRs) and 95% CIs from ORs.^25^ Gender differences in mental health were examined in models stratified by cohort. In the absence of a clear reference group in ethnic group comparisons (due to the diversity of the sample), the whole sample prevalence was used as the reference. Adjusted RRs are presented throughout. Unadjusted RRs are presented in online supplemental S1. Sample weights were calculated using 2016/2017 data from the National Pupil Database. Partially observed variables (SDQ (missing n, 106), SMFQ (missing n, 396), GAD-7 (missing n, 243), self-harm (missing n, 474), FSM (missing n, 240)) were multiply imputed in a multilevel model.^26^ Sensitivity analyses: (1) depression, anxiety and self-harm questions were not administered at two (originally pilot) schools. To assess any impact of including these two schools in analyses of SDQ (but not other outcome) data, we repeated analyses excluding these two schools (n=818); (2) we conducted a complete case analysis to assess the impact of missing data.

10.1136/jech-2020-214315.supp1 Supplementary data

## Results

### Participant characteristics

Of 4945 eligible students, 4353 (88.0%) participated at baseline and are included in this analysis. Reasons for non-participation were: persistent absence despite repeated visits by researchers (n=354, 7.2%); did not have parental consent (n=167, 3.4%); did not assent (n=57, 1.2%); and insufficient data due to technical issues (n=15, 0.03%). Just under a quarter (23.7%) of participants received FSM and 85% were from minority ethnic groups. Demographic characteristics were similar across cohorts, except for a higher proportion of British white students in cohort 1 (17.9%) compared with cohorts 2 (13.0%) and 3 (14.7%) (χ^2^=40.3, df=18, p=0.002). The REACH sample is highly representative of the target population; weighting made little difference to our estimates (online supplemental S2).

### Mental health problems, overall

The weighted prevalence was 18.6% (95% CI 16.4% to 20.8%) for mental health problems, 14.5% (11.8% to 17.2%) for depression, 13.7% (10.9% to 16.6%) for anxiety and 14.5% (12.4% to 16.6%) for lifetime self-harm (table 2). Of those with mental health problems, 69.0% were categorised as having problems on at least one other mental health measure.

**Table 2:** Weighted prevalence of mental health problems (and 95% CIs), overall and by group

	Probable mental health problems		Probable depression		Moderate-to-severe anxiety		Lifetime self-harm	
	% (95% CI)	Adj. RR (95% CI)	% (95% CI)	Adj. RR (95% CI)	% (95% CI)	Adj. RR (95% CI)	% (95% CI)	Adj. RR (95% CI)
All	18.6 (16.4 to 20.8)	–	14.5 (11.8 to 17.2)	–	13.7 (10.9 to 16.6)	–	14.5 (12.4 to 16.6)	–
Gender								
Boys	16.0 (14.1 to 17.8)	1	11.5 (9.3 to 13.8)	1	8.8 (6.3 to 11.3)	1	12.2 (10.0 to 14.3)	1
Girls	21.2 (18.7 to 23.8)	1.33 (1.18 to 1.48)	17.5 (14.3 to 20.7)	1.52 (1.30 to 1.73)	18.5 (16.2 to 20.8)	2.09 (1.58 to 2.59)	16.9 (13.9 to 19.8)	1.40 (1.06 to 1.75)
Cohort (school year group)								
1 (Y7)	19.2 (16.6 to 21.7)	1	13.5 (10.2 to 16.7)	1	13.0 (9.8 to 16.1)	1	12.7 (9.6 to 15.8)	1
2 (Y8)	18.8 (15.9 to 21.6)	0.97 (0.79 to 1.14)	16.4 (13.0 to 19.9)	1.22 (0.92 to 1.53)	14.7 (9.6 to 19.9)	1.12 (0.71 to 1.53)	16.4 (13.0 to 19.8)	1.29 (0.90 to 1.68)
3 (Y9)	17.7 (15.1 to 20.3)	0.91 (0.80 to 1.02)	13.7 (10.3 to 17.0)	1.02 (0.75 to 1.28)	13.6 (10.5 to 16.8)	1.03 (0.71 to 1.35)	14.8 (10.6 to 19.0)	1.17 (0.73 to 1.61)
Eligible for free school meals								
No	17.4 (14.9 to 19.9)	1	14.1 (11.1 to 17.0)	1	14.0 (11.1 to 16.9)	1	13.8 (11.4 to 16.1)	1
Yes	22.5 (19.8 to 25.2)	1.28 (1.05 to 1.51)	16.1 (12.3 to 19.8)	1.15 (0.84 to 1.46)	12.6 (8.6 to 16.5)	0.87 (0.64 to 1.10)	17.4 (14.2 to 20.5)	1.29 (1.02 to 1.56)
Ethnic group								
All	18.6 (16.4 to 20.8)	1	14.5 (11.8 to 17.2)	1	13.7 (10.9 to 16.6)	1	14.5 (12.4 to 16.6)	1
Black African	17.2 (14.1 to 20.4)	0.93 (0.80 to 1.07)	13.7 (10.7 to 16.7)	0.96 (0.81 to 1.12)	11.9 (9.1 to 14.7)	0.88 (0.73 to 1.05)	12.1 (7.9 to 16.2)	0.84 (0.70 to 0.99)
Black Caribbean	20.8 (16.8 to 24.8)	1.12 (0.94 to 1.31)	15.8 (10.3 to 21.2)	1.08 (0.89 to 1.30)	12.5 (8.0 to 16.9)	0.89 (0.71 to 1.11)	15.2 (11.9 to 18.5)	1.04 (0.85 to 1.25)
Other black	19.4 (15.1 to 23.7)	1.04 (0.67 to 1.54)	14.3 (9.8 to 18.8)	0.94 (0.55 to 1.51)	14.1 (8.1 to 20.1)	1.00 (0.57 to 1.62)	16.6 (9.2 to 24.0)	1.16 (0.72 to 1.78)
Mixed white and black	22.4 (16.6 to 28.3)	1.19 (0.94 to 1.47)	14.0 (8.8 to 19.3)	0.96 (0.72 to 1.26)	13.7 (8.5 to 19.0)	1.02 (0.75 to 1.35)	15.3 (9.9 to 20.7)	1.04 (0.78 to 1.35)
Other mixed ethnic groups	25.4 (19.3 to 31.5)	1.34 (1.02 to 1.74)	15.7 (11.5 to 20.0)	1.07 (0.75 to 1.48)	21.2 (15.6 to 26.9)	1.57 (1.15 to 2.08)	18.4 (13.9 to 22.9)	1.25 (0.90 to 1.69)
Indian, Pakistani, Bangladeshi	12.3 (6.8 to 17.8)	0.67 (0.42 to 1.01)	14.5 (7.7 to 21.4)	1.01 (0.66 to 1.48)	10.6 (5.6 to 15.6)	0.74 (0.43 to 1.19)	13.3 (6.9 to 19.6)	0.93 (0.60 to 1.39)
Latin American	18.8 (14.2 to 23.4)	1.04 (0.74 to 1.41)	16.1 (8.2 to 24.1)	1.10 (0.76 to 1.54)	18.2 (11.1 to 25.2)	1.35 (0.95 to 1.86)	15.7 (9.8 to 21.7)	1.07 (0.74 to 1.50)
White British	17.9 (13.2 to 22.7)	0.98 (0.81 to 1.17)	15.6 (12.0 to 19.3)	1.09 (0.89 to 1.32)	16.2 (10.9 to 21.5)	1.20 (0.97 to 1.45)	14.9 (12.6 to 17.1)	1.05 (0.85 to 1.27)
Non-British white	17.4 (13.3 to 21.5)	0.97 (0.76 to 1.22)	13.7 (9.4 to 18.0)	0.95 (0.71 to 1.23)	13.1 (9.1 to 17.1)	0.95 (0.70 to 1.25)	17.2 (12.3 to 22.1)	1.22 (0.95 to 1.54)
Any other/unknown	16.9 (12.4 to 21.5)	0.93 (0.69 to 1.22)	12.2 (7.6 to 16.7)	0.86 (0.61 to 1.19)	12.6 (8.1 to 17.1)	0.99 (0.70 to 1.36)	13.6 (8.1 to 19.2)	0.93 (0.66 to 1.27)

RRs adjusted for gender, free school meal eligibility, gender and ethnic group, as applicable. All estimates adjusted for clustering at school level.

RR, risk ratio.;

### Mental health problems, by group

#### Gender

Overall, prevalence was higher among girls than among boys for mental health problems (aRR 1.33, 95% CI 1.18 to 1.48), depression (1.52, 1.30 to 1.73), moderate-to-severe anxiety (2.09, 1.58 to 2.59) and lifetime self-harm (1.40, 1.06 to 1.75) (table 2). Mean internalising score was also higher among girls (6.1, 95% CI 5.9 to 6.4) compared with boys (5.0, 4.8 to 5.3), as were mean GAD (5.1, 4.7 to 5.5 vs 3.3, 2.9 to 3.7) and SMFQ (6.0, 5.4 to 6.6 vs 4.4, 4.0 to 4.9) scores (table 3).

**Table 3:** Weighted mean mental health scores (and 95% CIs), overall and by group

	SDQ total difficulties score	SDQ internalising score	SDQ externalising score	SMFQ score	GAD-7 score
	Mean (95% CI)	Mean (95% CI)	Mean (95% CI)	Mean (95% CI)	Mean (95% CI)
All	12.1 (11.7 to 12.5)	5.6 (5.3 to 5.8)	6.6 (6.3 to 6.8)	5.2 (4.7 to 5.7)	4.2 (3.7 to 4.7)
Gender					
Boys	11.7 (11.4 to 12.1)	5.0 (4.8 to 5.3)	6.8 (6.5 to 7.0)	4.4 (4.0 to 4.9)	3.3 (2.9 to 3.7)
Girls	12.5 (11.9 to 13.0)	6.1 (5.9 to 6.4)	6.3 (6.0 to 6.7)	6.0 (5.4 to 6.6)	5.1 (4.7 to 5.5)
Cohort (school year group)					
1 (Y7)	12.1 (11.6 to 12.5)	5.6 (5.3 to 5.8)	6.5 (6.2 to 6.8)	5.0 (4.4 to 5.5)	4.0 (3.6 to 4.5)
2 (Y8)	12.2 (11.8 to 12.6)	5.7 (5.4 to 6.0)	6.6 (6.4 to 6.8)	5.7 (5.1 to 6.2)	4.4 (3.7 to 5.1)
3 (Y9)	12.0 (11.5 to 12.5)	5.5 (5.2 to 5.8)	6.6 (6.3 to 6.8)	5.1 (4.5 to 5.7)	4.2 (3.6 to 4.8)
Eligible for free school meals					
No	12.0 (11.5 to 12.4)	5.5 (5.2 to 5.8)	6.5 (6.3 to 6.7)	5.1 (4.6 to 5.7)	4.2 (3.7 to 4.7)
Yes	12.6 (12.0 to 13.1)	5.8 (5.5 to 6.1)	6.8 (6.5 to 7.2)	5.6 (4.9 to 6.3)	4.2 (3.6 to 4.9)
Ethnic group					
Black African	11.6 (11.1 to 12.2)	5.2 (4.9 to 5.6)	6.4 (6.1 to 6.8)	5.0 (4.4 to 5.5)	3.8 (3.3 to 4.3)
Black Caribbean	12.8 (12.1 to 13.5)	5.3 (4.9 to 5.7)	7.4 (7.0 to 7.8)	5.6 (4.6 to 6.5)	4.0 (3.1 to 5.0)
Other black	12.3 (11.4 to 13.2)	5.3 (4.7 to 6.0)	7.0 (6.5 to 7.5)	4.6 (3.9 to 5.3)	3.9 (3.0 to 4.7)
Mixed white and black	12.7 (11.8 to 13.7)	5.5 (5.0 to 6.1)	7.2 (6.6 to 7.8)	5.0 (4.3 to 5.8)	4.0 (3.4 to 4.7)
Other mixed ethnic groups	13.2 (12.4 to 13.9)	6.3 (5.8 to 6.7)	6.9 (6.5 to 7.3)	5.7 (4.9 to 6.4)	5.3 (4.4 to 6.2)
Indian, Pakistani, Bangladeshi	10.9 (10.1 to 11.8)	5.2 (4.7 to 5.8)	5.7 (5.2 to 6.1)	5.1 (4.1 to 6.0)	3.9 (3.0 to 4.7)
Latin American	12.8 (12.2 to 13.4)	6.2 (5.7 to 6.7)	6.6 (5.9 to 7.4)	5.7 (4.2 to 7.1)	4.6 (3.6 to 5.5)
White British	12.0 (11.5 to 12.6)	6.0 (5.6 to 6.3)	6.1 (5.8 to 6.4)	5.6 (5.0 to 6.2)	4.8 (4.1 to 5.6)
Non-British white	11.9 (11.2 to 12.6)	5.7 (5.2 to 6.3)	6.2 (5.7 to 6.6)	5.0 (4.2 to 5.9)	4.3 (3.7 to 4.9)
Any other/unknown	11.9 (11.1 to 12.7)	5.7 (5.3 to 6.2)	6.2 (5.7 to 6.8)	4.9 (4.2 to 5.7)	4.0 (3.3 to 4.8)

GAD, Generalised Anxiety Disorder Scale; SDQ, Strength and Difficulties Questionnaire; SMFQ, Short Mood and Feelings Questionnaire.

#### Cohort

There was no clear evidence for variations in prevalence of mental health problems by cohort.

#### Gender and cohort

**Figure 1:**
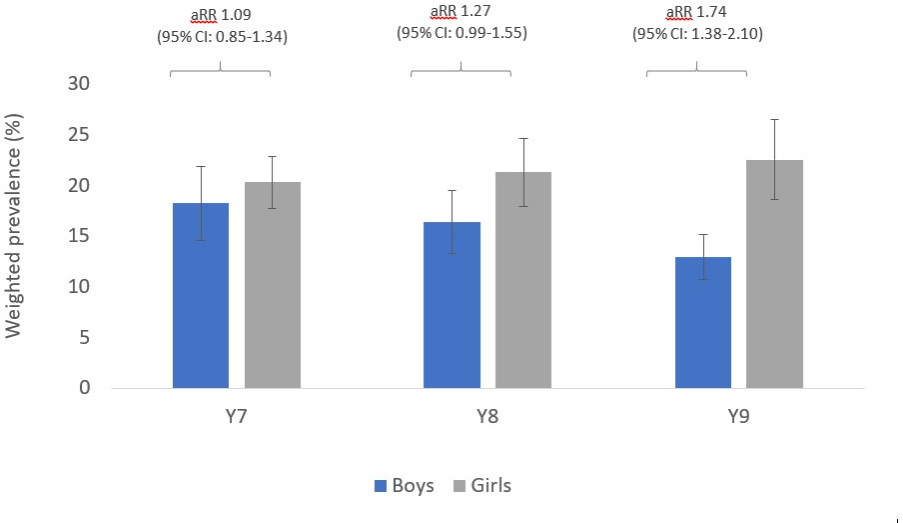
Prevalence of probable mental health problems (and 95% CIs), by gender and cohort. aRR, adjusted risk ratio.

In models stratified by cohort (figure 1), clear patterns emerged of variation in gender differences by cohort. Prevalence of mental health problems was similar for boys and girls in cohort 1 (year 7), but higher among girls in cohort 2 (year 8) and higher still in cohort 3 (year 9). For example, the relative risks of mental health problems among girls (vs boys) were 1.09 (95% CI 0.85 to 1.34), 1.27 (0.99 to 1.55) and 1.74 (1.38 to 2.10) in cohorts 1–3, respectively (figure 1). A similar pattern was observed for depression, anxiety, lifetime self-harm (online supplemental S3) and internalising scores (online supplemental S4). By contrast, mean externalising scores were similar between boys and girls in each cohort (online supplemental S4).

#### Socioeconomic disadvantage

Among those receiving (vs not receiving) FSM, risks of mental health problems (aRR 1.28, 95% CI 1.05 to 1.51) and of lifetime self-harm (1.29, 1.02 to 1.56) were around 30% higher (table 2). However, risks of anxiety and depression were similar between the two groups.

#### Ethnic group

Overall, there were many similarities, with some variations, in prevalence of mental health problems by ethnic group (table 2). For example, compared with the overall sample, mental health problems were more common among those from other mixed ethnic backgrounds (aRR 1.34, 95% CI 1.02 to 1.74) and less common among those from Indian/Pakistani/Bangladeshi backgrounds (0.67, 0.42 to 1.01), but otherwise similar across ethnic groups. A similar pattern was observed for anxiety, although there was also some evidence for higher prevalence of anxiety among those from Latin American (1.35, 0.95 to 1.86) and non-British white (1.20, 0.97 to 1.35) backgrounds compared with the overall sample. We found no evidence of differences in prevalence of depression by ethnic group (14%–16% across all groups). For self-harm, risk was lower among those from black African backgrounds compared with the overall sample (0.84, 0.70 to 0.99), and there was some evidence for increased risk among those from other mixed ethnic backgrounds (1.25, 0.90 to 1.69), but otherwise there was little difference across groups. Overall, prevalence of mental health problems was generally similar between those from black African, black Caribbean and white British backgrounds (table 2), but there was evidence for variations in externalising scores between these groups. For example, mean externalising scores were higher among black Caribbean (7.4, 7.0 to 7.8), mixed black and white (7.2, 6.6 to 7.8) and other Black (7.0, 6.5 to 7.5) and lower among British white (6.1, 5.8 to 6.4) (table 3).

### Sensitivity analyses

The findings were not substantively changed in sensitivity analyses.

## Discussion

In the most recent and comprehensive study of adolescent mental health in an inner city area in the UK, we found that mental health problems were notably more common than reported nationally; around one in five had a mental health problem compared with around one in seven to eight nationally. This equates to, on average, 6 pupils in a class of 30 in inner city schools. We also found that differences between boys and girls emerge in older cohorts, suggesting this may be a critical point at which lifelong differences emerge, and we found interesting similarities, with some variations, by ethnic group and socioeconomic status.

### Methodological considerations

Several methodological issues should be noted when considering our findings. First, in this part of REACH, we used self-report measures of mental health. These measures are not designed to provide clinical diagnoses; they are screening tools designed to identify those likely to have a diagnosable problem. It is likely, then, that some young people in our study are misclassified. Nonetheless, the measures used in our study were developed/validated for this age group, are widely used in epidemiological studies of adolescent mental health, and our use of multiple screening measures (SDQ, SMFQ, GAD-7) provides more detailed information on participants’ mental health compared with many other studies.^8 11^ Collection of more in-depth information via interviews with a nested subsample is ongoing and will enable triangulation of data.

Second, those who did not participate were primarily those who were persistently absent from school. This is a potential source of bias, but the impact, if any, would be conservative estimates—that is, underestimation of the prevalence of, and many subgroup differences in, mental health problems—because those who did not participate may be more likely to have problems and disadvantaged backgrounds compared with those who did. Moreover, our cohorts are highly representative of the target population, weighting made little difference to our estimates, and we achieved high participation rates: 88% in REACH compared with, for example, 52% in the MHCYP.^7^

### The nature and extent of adolescent mental health problems

Estimates of the prevalence of mental health problems among adolescents vary across studies. Previous national studies in the UK estimate a prevalence of around 10%–14%, with, overall, little change among secondary school-aged pupils over time.^7 8 27^ Some of this variation may be due to the methods used. For instance, in the MHCYP (2017, 11–16 years), the estimated prevalence was 14% using multi-informant interviews.^7^ Understanding Society (2009/2010 and 2011/2012, 10–15 years) estimated 12% using similar methods to ours.^8^ Nonetheless, these differences are small. Our estimate, one in five (19%), is notably higher, and strongly suggests higher prevalence in inner cities. Further, it may be that in diverse inner city contexts prevalence has increased, more so than reported nationally, over the past 15–20 years. Our estimates (16% among boys, 21% among girls; 11–14 years) are notably higher than observed in two similar multiethnic inner city London studies conducted in 2001 (boys, 10%; girls 12%; 11–13 years^9^) and 2003 (boys, 14%; girls, 17%; 11–14 years^10^). Taken together—while noting the limitations of directly comparing these data—the evidence suggests that (1) problems are more common among adolescents in inner city London compared with national samples, and (2) within inner city London, prevalence has increased over the last 15–20 years.

However, while our data suggest the prevalence of mental health problems may, overall, be elevated in inner city London compared with nationally, prevalence of (self-report) depression and self-harm appear to be similar to, or slightly lower than, recent national estimates (around 15% for lifetime self-harm and 16% for depression).^27 28^ It is possible, then, that elevated risk in inner cities is driven by externalising/behavioural problems rather than internalising/emotional problems. Directly comparable data on externalising problems in this age group are scarce and further research is required, but this observation may reflect substantive differences in the experiences of young people growing up in diverse inner city areas, and/or variations in the manifestation of distress among diverse groups. These findings are important and reinforce the need for localised studies of mental health.^16^ Nationally, there is a strong focus on addressing rising emotional problems among girls and young women; much less attention is directed at externalising problems, or indeed emotional problems and self-harm among boys. That 12% of boys in inner city London have self-harmed by age 11–14 is concerning and there is an urgent need for greater understanding and preventive strategies.

Consistent with existing evidence,^7 29^ mental health problems were more common among girls than among boys. However, our data—which permit more fine-grained analyses than is often possible in other surveys—suggest risk of problems is similar between boys and girls in year 7 and becomes more pronounced with age. These data, at this stage, are cross-sectional, so cohort effects cannot be ruled out, but this observation mirrors findings from previous research in London^10^ and internationally,^30^ and, if replicated with longitudinal REACH data, may have significant implications. Irrespective of whether gender differences at this age reflect genuine differences in risk, or under-reporting of distress among boys, these findings point to early adolescence as the possible point at which lifelong gender inequalities in (reported) mental health problems first emerge.

Our data—the most recent and detailed information on adolescent mental health among diverse groups and one of few studies with sufficient power for ethnic group comparisons—suggests prevalence of mental health problems is lower among Indian/Bangladeshi/Pakistani adolescents, and higher among those from other mixed ethnic backgrounds (a group that is growing in size in the UK), but otherwise similar across ethnic groups. This observation is broadly consistent with earlier findings.^9–11^ Given that most UK minority ethnic groups experience disproportionately high levels of adversity (eg, discrimination, poverty), these observations are striking.

### Implications

Our findings suggest important implications for research, prevention/intervention and local services and policy. First, national data are important but of limited use in understanding the extent and nature of distress in local communities; localised studies are needed to inform prevention strategies and local service provision.^16^ Second, the widely reported gender difference in mental health likely emerges during the first few years of secondary school; this may be a critical period for prevention and intervention. Third, the many similarities in prevalence of mental health problems across ethnic and socioeconomic groups are striking and understanding these similarities should be a priority for future research; it will advance our understanding of resilience and protective factors, and therefore how to promote mental health and prevent problems, in young people from all backgrounds.

**What is already known on this subject**

- Recent estimates from national surveys in England suggest that around one in seven to eight adolescents have a mental health problem. While national surveys provide useful information on the prevalence of mental health problems nationally, they tend not to provide detailed information on specific groups in which prevalence (and change in prevalence over time) may differ from the national average, for example, minority ethnic and socially disadvantaged groups living in densely populated inner city areas. At present, there is very little up-to-date information on the extent and nature of problems among young people from diverse groups and inner city areas in the UK.

**What this study adds**

- Resilience, Ethnicity and AdolesCent Mental Health is an accelerated cohort study of 4353 young people (age 11–14 years at baseline; 85% from minority ethnic groups, one in four receiving free school meals) attending secondary schools in inner city London. In analyses of baseline data, we found that around one in five (19%) experienced recognisable mental health issues in the 6 months prior to assessment, a notably higher prevalence than in recent national estimates (10%–14%) and estimates for inner city London from around 15–20 years ago (10%–12%). Around one in seven (14%) reported lifetime self-harm. Mental health problems were more common among girls than boys, a difference that was more pronounced in older cohorts, and among those from economically disadvantaged backgrounds. We observed many similarities—with some variations—in the prevalence of mental health problems across ethnic groups, with highest prevalence (around one in four) being among those of mixed ethnic backgrounds (25%). These findings underscore the importance of local population-based studies to more fully characterise and understand variations in prevalence, and in the distribution of risk and protective factors, by area and social group and to better inform local strategies for prevention and intervention. The many similarities in prevalence of mental health problems across ethnic and socioeconomic groups are striking and understanding these similarities should be a priority for future research; it will advance our understanding of resilience and protective factors, and thereforehow to promote mental health and prevent problems, in young people from all backgrounds.

## Data Availability

Data are available on reasonable request. We welcome and encourage requests from researchers wishing to access REACH data for specific research projects or collaborations. Information about our data access policy, which aims to make REACH data as accessible as possible while adhering to legal and ethical principles and protecting the privacy of schools and participants, can be requested from the project coordinator (gemma.knowles@kcl.ac.uk) (and will be available on our website in due course: www.thereachstudy.com). Further information about REACH, including the study protocol, planned analyses, and the available data, can be found here: https://www.thereachstudy.com/information-for-researchers.html.
