# Supplementary material for: Mental distress among young people in inner cities: the Resilience, Ethnicity and AdolesCent Mental Health (REACH) study
Source: J Epidemiol Community Health. 2021 Feb 8;75(6):515–22. doi: 10.1136/jech-2020-214315 (PMC8142438; doi:10.1136/jech-2020-214315)

Supplement S1

Table S1. Unadjusted risk ratios (and 95% confidence intervals) for mental health problems

	Probable mental health problems<br>RR (95% CI)	Probable depression<br>RR (95% CI)	Moderate-to-severe anxiety<br>RR (95% CI)	Lifetime self-harm<br>RR (95% CI)
**Gender**				
Boys	1	1	1	1
Girls	**1.33 (1.18, 1.48)**	**1.52 (1.32, 1.73)**	**2.11 (1.61, 2.61)**	**1.39 (1.06, 1.72)**
**Cohort (school year group)**				
1 (Y7)	1	1	1	1
2 (Y8)	0.98 (0.81, 1.15)	1.22 (0.93, 1.52)	1.14 (0.72, 1.56)	1.29 (0.91, 1.67)
3 (Y9)	0.92 (0.82, 1.03)	1.02 (0.75, 1.28)	1.05 (0.73, 1.38)	1.16 (0.74, 1.58)
**Eligible for free school meals**				
No	1	1	1	1
Yes	**1.30 (1.07, 1.53)**	1.14 (0.85, 1.44)	0.89 (0.66, 1.13)	**1.27 (1.01, 1.52)**
**Ethnic group**				
All	1	1	1	1
Black African	0.93 (0.80, 1.07)	0.94 (0.80, 1.11)	0.86 (0.72, 1.02)	**0.83 (0.70, 0.98)**
Black Caribbean	1.12 (0.95, 1.32)	1.09 (0.90, 1.31)	0.91 (0.73, 1.12)	1.05 (0.86, 1.26)
Other black	1.06 (0.69, 1.56)	0.98 (0.58, 1.55)	1.03 (0.61, 1.63)	1.14 (0.71, 1.74)
Mixed white and black	1.20 (0.96, 1.49)	0.97 (0.72, 1.26)	1.00 (0.74, 1.31)	1.05 (0.80, 1.36)
Other mixed ethnic groups	**1.36 (1.04, 1.75)**	1.08 (0.76, 1.49)	**1.54 (1.14, 2.03)**	1.28 (0.93, 1.72)
Indian, Pakistani, Bangladeshi	**0.65 (0.41, 0.99)**	0.99 (0.65, 1.46)	0.77 (0.46, 1.19)	0.92 (0.59, 1.36)
Latin American	1.02 (0.73, 1.38)	1.12 (0.78, 1.55)	1.31 (0.93, 1.79)	1.08 (0.75, 1.51)
White British	0.97 (0.80, 1.16)	1.08 (0.88, 1.31)	1.18 (0.97, 1.42)	1.02 (0.83, 1.25)
Non-British white	0.93 (0.73, 1.18)	0.95 (0.72, 1.23)	0.96 (0.72, 1.26)	1.18 (0.92, 1.49)
Any other/Unknown	0.91 (0.67, 1.19)	0.84 (0.59, 1.16)	0.91 (0.65, 1.25)	0.93 (0.67, 1.27)

Supplement S2

Table S2. Unweighted prevalence (and 95% confidence intervals) of mental health problems, overall and by group.

	Probable mental health problems<br>% (95% CI)	Probable depression<br>% (95% CI)	Moderate-to-severe anxiety<br>% (95% CI)	Lifetime self-harm<br>% (95% CI)
All	18.8 (16.6, 20.9)	14.5 (11.9, 17.2)	13.9 (10.6, 17.2)	14.4 (12.2, 16.5)
**Gender**				
Boys	16.1 (14.0, 18.2)	11.6 (9.2, 14.1)	9.0 (7.1, 10.9)	12.3 (10.1, 14.5)
Girls	21.4 (18.9, 23.8)	17.3 (14.1, 20.5)	18.3 (15.3, 21.2)	16.3 (13.8, 18.9)
**Cohort (school year group)**				
1 (Y7)	19.4 (16.7, 22.1)	13.5 (10.5, 16.4)	13.1 (9.6, 16.5)	12.6 (10.1, 15.1)
2 (Y8)	18.7 (16.0, 21.4)	16.6 (13.0, 20.1)	15.2 (11.2, 19.1)	16.3 (13.2, 19.5)
3 (Y9)	18.0 (15.3, 20.8)	13.8 (10.6, 17.0)	13.7 (9.9, 17.4)	14.8 (11.8, 17.7)
**Eligible for free school meals**				
No	17.6 (15.5, 19.7)	14.1 (11.4, 16.8)	14.2 (10.8, 17.5)	13.6 (11.4, 15.8)
Yes	22.4 (19.1, 25.6)	16.0 (12.2, 19.8)	12.8 (9.0, 16.6)	17.3 (13.8, 20.8)
**Ethnic group**				
Black African	17.3 (14.5, 20.1)	13.6 (10.4, 16.9)	11.9 (8.5, 15.3)	12.0 (9.3, 14.7)
Black Caribbean	20.9 (17.3, 24.5)	15.9 (11.6, 20.3)	12.8 (8.6, 16.9)	15.3 (11.5, 19.1)
Other black	20.1 (12.9, 27.3)	14.4 (7.2, 21.5)	14.5 (7.3, 21.8)	17.5 (10.0, 25.0)
Mixed white and black	22.8 (18.1, 27.5)	14.1 (9.4, 18.7)	14.0 (9.0, 19.0)	15.5 (10.9, 20.1)
Other mixed ethnic groups	25.8 (19.7, 31.8)	15.9 (10.2, 21.6)	21.0 (14.1, 27.9)	17.9 (12.2, 23.6)
Indian, Pakistani, Bangladeshi	12.5 (7.5, 17.6)	15.6 (8.8, 22.3)	10.9 (5.3, 16.6)	13.5 (7.6, 19.4)
Latino/Latina	18.7 (13.2, 24.1)	16.2 (9.7, 22.7)	18.0 (10.8, 25.1)	16.3 (10.0, 22.6)
British white	17.3 (13.8, 20.8)	15.4 (11.4, 19.3)	15.7 (11.2, 20.3)	14.1 (10.6, 17.5)
Non-British white	17.5 (13.4, 21.6)	13.6 (9.0, 18.2)	13.1 (8.3, 17.9)	16.8 (12.0, 21.5)
Any other/Unknown	17.1 (12.5, 21.7)	12.4 (7.6, 17.1)	13.1 (8.0, 18.3)	13.4 (8.7, 18.0)

Supplement S3

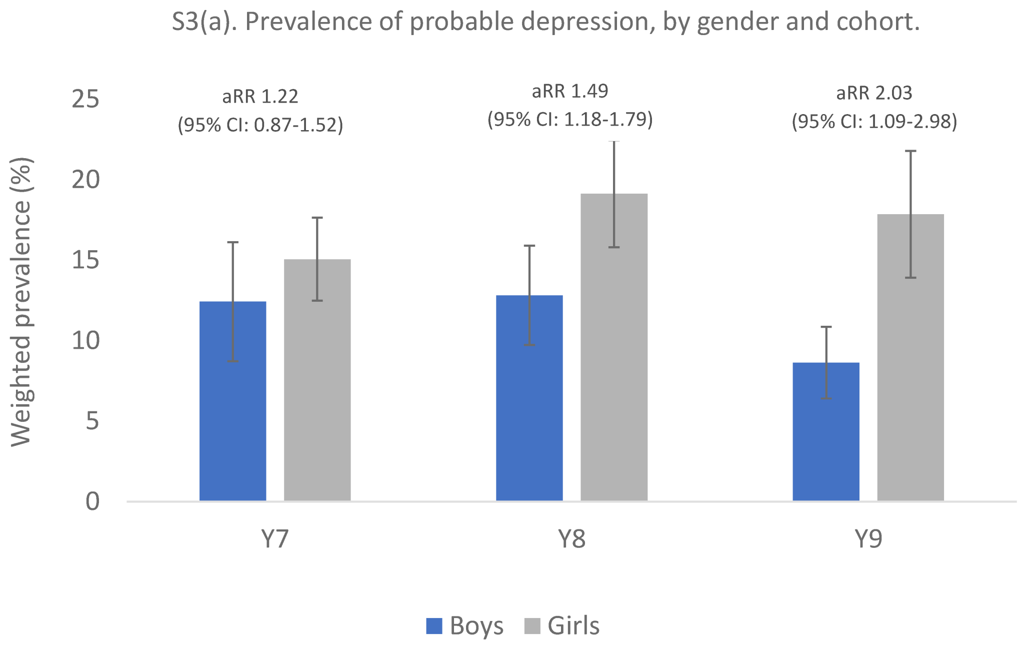

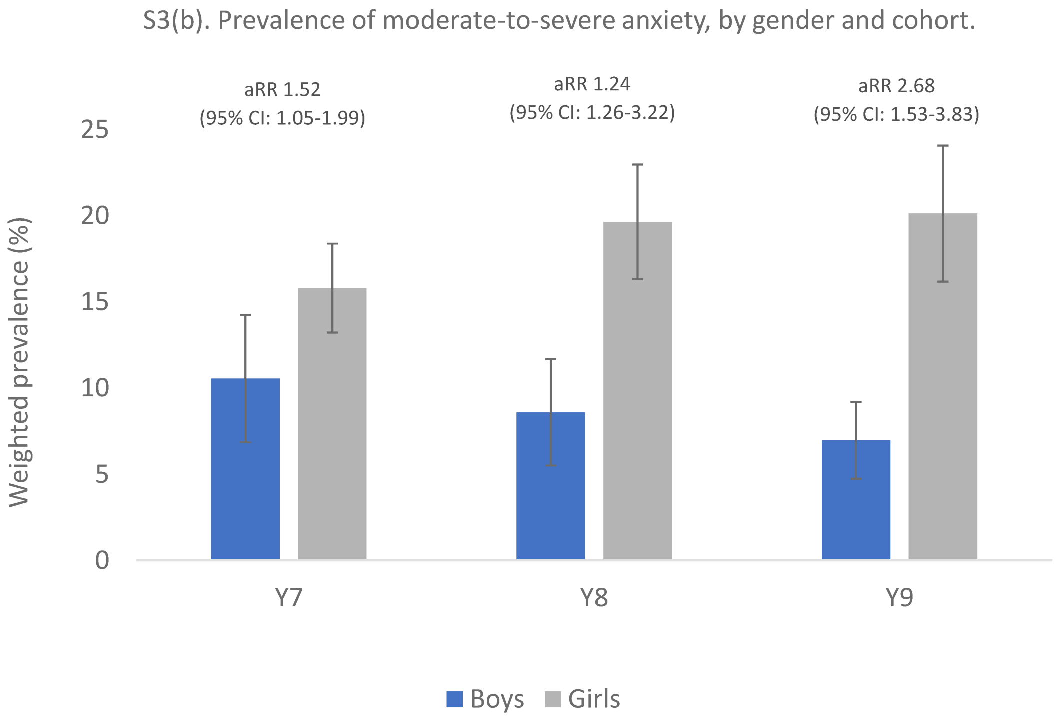

S3(c). Prevalence of lifetime self-harm, by gender and cohort.

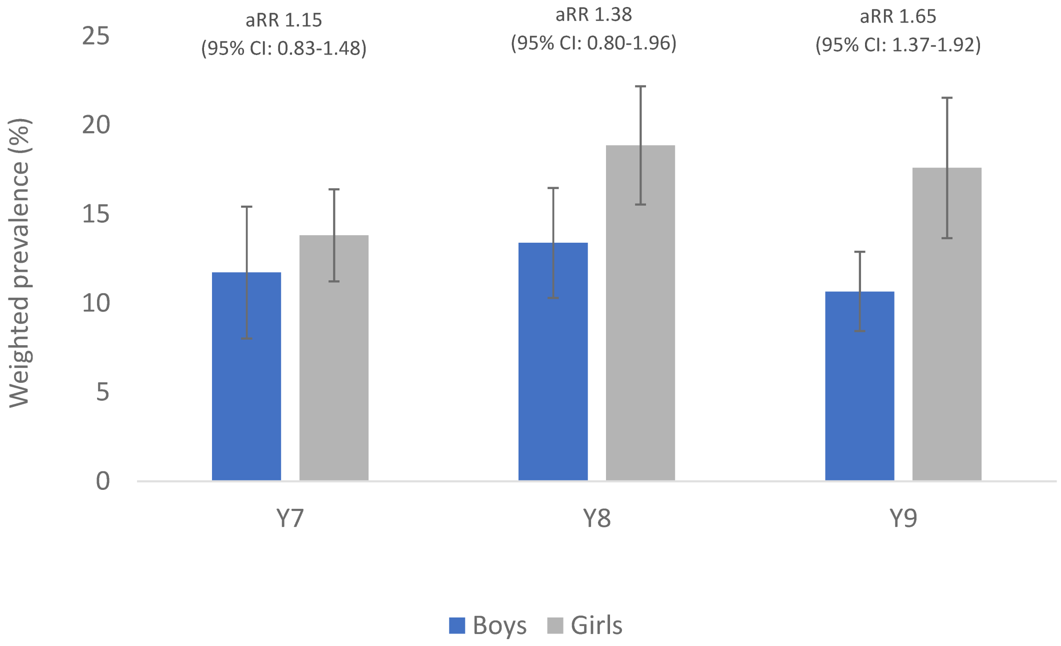

Supplement S4

S4(a). Weighted mean internalising scores (and 95% CIs), by gender and cohort

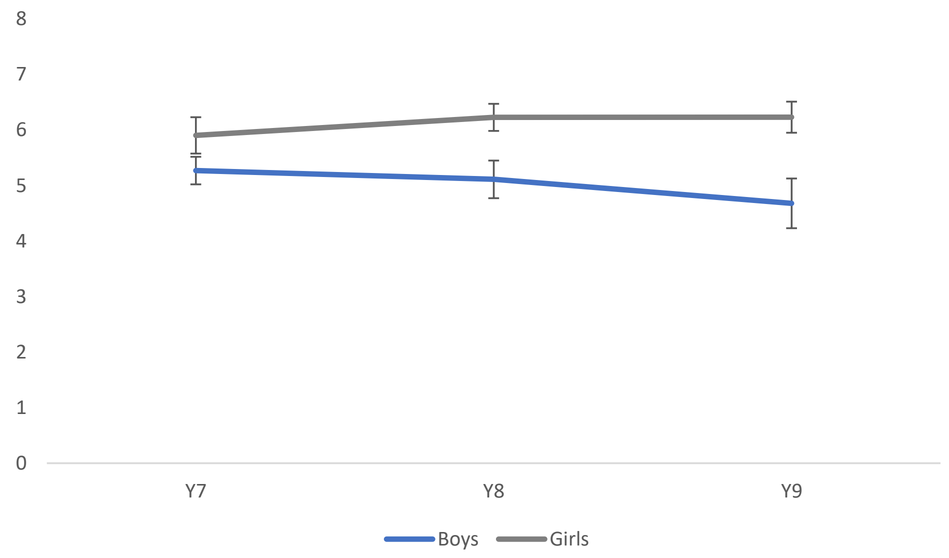

S4(b). Weighted mean externalising scores (and 95% CIs) , by gender and cohort

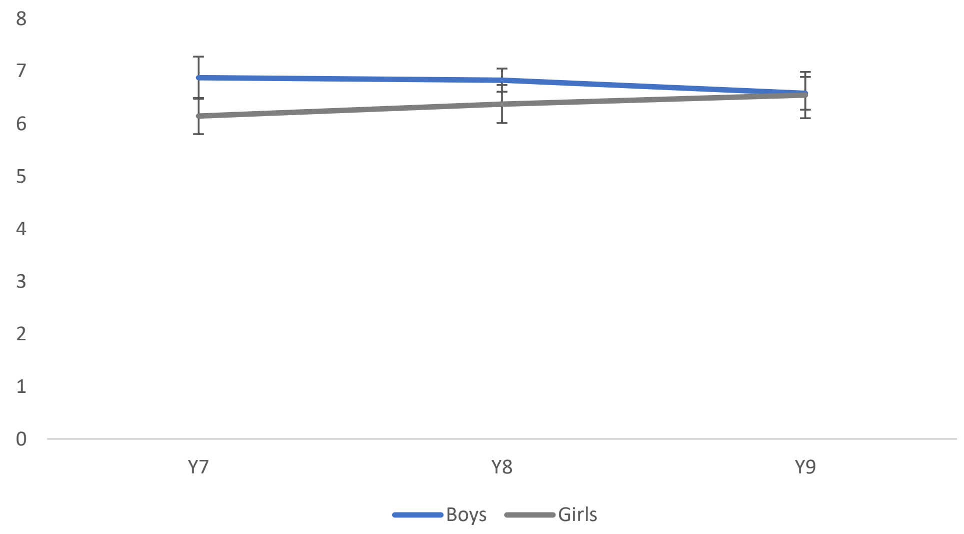

Supplement: Supplementary data [file jech-2020-214315supp001.pdf]
